# Training types associated with knowledge and experience in public health workers

**DOI:** 10.1186/s13690-022-00788-4

**Published:** 2022-01-27

**Authors:** Zui Narita, Yoshio Yamanouchi, Kazuo Mishima, Yoko Kamio, Naoko Ayabe, Ryoko Kakei, Yoshiharu Kim

**Affiliations:** 1 Department of Behavioral Medicine, National Institute of Mental Health, National Center of Neurology and Psychiatry, 4-1-1 Ogawahigashi, Kodaira, Tokyo, 187-8553 Japan; 2 Aisei Century Hospital, Nagoya, Aichi Japan; 3 Department of Neuropsychiatry, Akita University Graduate School of Medicine, Akita, Japan; 4 Department of Sleep-Wake Disorders, National Institute of Mental Health, National Center of Neurology and Psychiatry, Tokyo, Japan; 5 Department of Preventive Intervention for Psychiatric Disorders, National Institute of Mental Health, National Center of Neurology and Psychiatry, Tokyo, Japan; 6 Institute of Education and Human Development, Ochanomizu University, Tokyo, Japan; 7 Department of Regional Studies and Humanities, Faculty of Education and Human Studies, Akita University, Akita, Japan; 8 Wayo Women’s University, Chiba, Japan

**Keywords:** Training course, Community mental health, Public health, Primary care

## Abstract

**Background:**

Training non-specialist workers in mental healthcare improves knowledge, attitude, confidence, and recognition of mental illnesses. However, still little information is available on which type of mental health training is important in the improvement of these capacities.

**Methods:**

We studied web-based survey data of 495 public health workers to examine training types associated with knowledge and experience in supporting individuals with mental illness. Multivariable logistic regression analysis was conducted to evaluate the association between a lack of knowledge and experience (outcome) and mental health training (exposure). We fitted three regression models. Model 1 evaluated unadjusted associations. Model 2 adjusted for age and sex. Model 3 adjusted for age, sex, years of experience, mental health full-time worker status, and community population. Bias-corrected and accelerated bootstrap confidence intervals (CIs) were used.

**Results:**

For all training types, the association between a lack of knowledge and experience and mental health training attenuated as the model developed. In Model 3, a lack of knowledge and experience was significantly associated with training in specific illness (OR, 0.54; 95% CI, 0.32–0.93) and screening and assessment (OR, 0.63; 95% CI, 0.39–0.99). Non-significant results were produced for training in counseling, psychosocial support, collaborative work, and law and regulation in Model 3.

**Conclusions:**

We believe that the present study provides meaningful information that training in specific illness and screening and assessment may lead to knowledge and experience of public health workers. Further studies should employ a longitudinal design and validated measurements.

**Supplementary Information:**

The online version contains supplementary material available at 10.1186/s13690-022-00788-4.

## Background

The coronavirus disease 2019 (COVID-19) global pandemic highlights the importance of mental illness as one of the significant causes of disability and the global burden of disease [1–4]. Mental illness has the longest years lived with disability and is the same level as cardiovascular and circulatory diseases in disability-adjusted life-years [5]. Across all regions of the world, mental illness is highly prevalent and affecting individuals [6]. Approximately one-fifth of individuals in a general population experiences 12-month mental illness [6]. Previous data suggested that the global direct and indirect economic costs of mental illness were estimated at US$2.5 trillion [7]. Thus, adequate management of mental illness is crucial for social recovery in the post-COVID-19 era.

These decades have seen significant changes of mental health care in many countries worldwide [8]. Of these, the development of community-based care was one of the essential changes [8, 9]. In communities, non-specialist workers, such as primary care and public health workers, need to manage individuals with mental illness. Integrating mental health services at the primary care level is the most viable way to decrease the treatment gap and ensure that people undergo the mental health care they need [10].

Certain skills and competencies are required to assess, diagnose, treat, support, and refer individuals with mental illness. Therefore, non-specialist workers need to be adequately prepared and supported in their mental health work [10, 11]. The World Health Organization (WHO) Mental Health Action Plan (2013–2020) and the WHO Mental Health Gap Action Programme (mhGAP) recommended adequate training in non-specialist workers in diagnosing and treating mental illness [12, 13]. This is specifically of relevance in communities with small or previously non-existent budgets for mental health [14].

Previous studies with a pre-post design showed significant effects of mental health training on non-specialist workers’ knowledge, attitude, confidence, and recognition of mental illnesses [15–19]. Moreover, a cluster-randomized controlled trial showed that training of non-specialist workers improved the detection of mental illnesses [20]. These effects of mental health training were also verified by a systematic review including 29 studies [14]. Nevertheless, still little information is available on which type of mental health training is important in the improvement of such capacities. Japan reportedly has a poor quality of community mental health [21, 22], and such information is crucial to improve the mental health service. In the present study, we studied web-based survey data of 495 public health workers in Japan to examine training types associated with knowledge and experience in supporting individuals with mental illness.

## Methods

### Sample

We analyzed data from public health workers working at community health care centers in Japan who underwent a cross-sectional web-based survey. The survey was administered from 8 January to 28 February 2018 using SurveyMonkey software. Participants were recruited by the National Center of Neurology and Psychiatry. We mailed letters to managers of all 589 health care centers in Japan. Next, each manager distributed the letters to public health workers in the health care center. The letter showed the URL of the survey so that each participant could access it. Note that we only included certified individuals such as public health nurses or social workers. A total of 643 public health workers agreed to participate. Of these, 495 public health workers (77.0%) completed the survey. Details of participants’ professional backgrounds are shown in Supplementary Table S1. The survey was approved by the National Center of Neurology and Psychiatry Institutional Review Board (A2018–097). All participants provided written informed consent.

### Lack of knowledge and experience

Participants self-reported if they had a lack of knowledge and experience enough to support individuals with mental illness. They were asked to select one of seven response options regarding how much percentage of cases they had a lack of knowledge and experience: (1) 100–80%, (2) 79–60%, (3) 59–40%, (4) 39–20%, (5) seldom, (6) not at all, or (7) not sure. Endorsing (1), (2), or (3) was regarded as a lack of knowledge and experience.

### Mental health training

Participants self-reported which type of mental health training they underwent. They were asked to select all that apply from six response options: (1) specific illness, (2) screening and assessment, (3) counseling, (4) psychosocial support, (5) collaborative work, or (6) law and regulation. We included the components featured in the mhGAP Intervention Guide [23]. For instance, the mhGAP Intervention Guide recommends contacting legal resources in a situation of maltreatment, abuse, and neglect [23].

### Sociodemographic factors

Sociodemographic factors that may confound the relationship between each training and knowledge and experience were included in analyses as covariates. Participants self-reported sociodemographic factors, including age (29 years or younger, 30–39 years, 40–49 years, 50 years or older), sex, years of experience, mental health full-time worker status (yes/no), community population (50,000 or smaller, 50,000-200,000, 200,000-500,000, 500,000-1000,000, 1000,000 or larger). Years of experience and mental-health full-time worker status were adjusted for since they may be linked to a higher chance of having training and more knowledge and experience. Community population was included because the relationship to community individuals may be associated with training’s success [24]. Years of education were not adjusted for as all participants had at least a college degree.

### Statistical analysis

All statistical analyses were conducted by using R version 4.1.0. Baseline characteristics of participants who lacked knowledge and experience and those who did not were compared using independent-sample t-tests and chi-square tests. Multivariable logistic regression analysis was conducted to evaluate the association between a lack of knowledge and experience (outcome) and mental health training (exposure). We fitted three regression models. Model 1 evaluated unadjusted associations. Model 2 adjusted for age and sex. Model 3 adjusted for age, sex, years of experience, mental health full-time worker status, and community population. Bias-corrected and accelerated bootstrap confidence intervals (CIs) were used to acquire an accurate estimation [25–27]. In conducting bootstrap CIs, the size of the bootstrap sample was set at 1000 with 95% CIs [28]. The significance level was set at a *p*-value less than 0.05.

## Results

### Baseline characteristics

Table 1 shows the baseline characteristics of participants. A total of 308 participants (62.2%) reported a lack of knowledge and experience. Individuals with a lack of knowledge and experience showed fewer years of experience (*p* <  0.001), lower proportion of mental health full-time workers (*p* = 0.001), training in specific illness (*p* <  0.001), screening and assessment (*p* = 0.005), psychosocial support (*p* = 0.03), and law and regulation (*p* = 0.001). Two groups significantly differed in age (*p* <  0.001), i.e., individuals with a lack of knowledge and experience were more likely to be 39 years or younger and less likely to be 40 years or older. No significant difference was shown in sex, community population, and training in counseling and collaborative work. Training in specific illness was most frequently reported in both groups (68.8 and 82.3%, respectively). Details of training in specific illness are shown in Supplementary Table S2.

**Table 1:** Baseline characteristics of study participants

Characteristic	Overall (*n* = 495)	Lack of knowledge and experience		*P* value
		Yes (*n* = 308)	No (*n* = 187)	
Sex, No. (%)^a^				0.08
Male	87 (17.6)	47 (15.4)	40 (21.5)	
Female	405 (81.8)	259 (84.6)	146 (78.5)	
Age, No. (%)^b^				< 0.001
-29	91 (18.4)	77 (25.0)	14 (7.5)	
30–39	148 (29.9)	99 (32.1)	49 (26.3)	
40–49	138 (27.9)	78 (25.3)	60 (32.3)	
50-	117 (23.6)	54 (17.5)	63 (33.9)	
Years of experience, mean (SD), y	15.7 (10.3)	13.2 (9.6)	19.5 (10.3)	< 0.001
Mental health full-time worker, No. (%)^c^				=0.001
No	297 (60.0)	202 (66.2)	95 (51.4)	
Yes	193 (38.9)	103 (33.8)	90 (48.6)	
Community population, No. (%)^d^				
-50,000	176 (35.6)	120 (39.0)	56 (30.1)	0.09
50,000-200,000	168 (33.9)	106 (34.4)	62 (33.3)	
200,000-500,000	75 (15.2)	42 (13.6)	33 (17.7)	
500,000-1000,000	38 (7.7)	17 (5.5)	21 (11.3)	
1000,000-	36 (7.2)	22 (7.1)	14 (7.5)	
Training type, No. (%)				
Specific illness	365 (73.7)	212 (68.8)	153 (82.3)	< 0.001
Screening and assessment	127 (25.7)	72 (23.4)	55 (34.9)	0.005
Counseling	317 (64.0)	191 (62.0)	126 (67.7)	0.20
Psychosocial support	173 (34.9)	97 (31.5)	76 (40.9)	0.03
Collaborative work	226 (45.7)	135 (43.8)	91 (48.9)	0.27
Law and regulation	171 (34.5)	90 (29.2)	81 (43.5)	0.001

a Missing data for three participants

b Missing data for one participant

c Missing data for five participants

d Missing data for two participants

### Relationship between knowledge and experience and training types

Table 2 summarizes the results of multivariable logistic regression analyses. For all training types, the association between a lack of knowledge and experience and mental health training attenuated as the model developed (i.e., odds ratios were lowest in Model 1 and highest in Model 3). In Model 3, a lack of knowledge and experience was significantly associated with training in specific illness (OR, 0.54; 95% CI, 0.32–0.93) and screening and assessment (OR, 0.63; 95% CI, 0.39–0.99). Training in psychosocial support and law and regulation showed a statistical significance in Model 1 and Model 2, which disappeared in Model 3. Non-significant results were produced for training in counseling and collaborative work in all Model 1, Model 2, and Model 3.

**Table 2:** Associations between a lack of knowledge and experience and mental health training

	Lack of knowledge and experience Odds ratio [95% confidence interval]					
Model 1, unadjusted						
Training type						
Specific illness	0.48^**^ [0.30–0.75]					
Screening and assessment		0.57^**^ [0.37–0.86]				
Counseling			0.78 [0.53–1.15]			
Psychosocial support				0.67^*^ [0.45–0.98]		
Collaborative work					0.81 [0.56–1.19]	
Law and regulation						0.54^**^ [0.37–0.79]
Model 2, adjusted for age and sex						
Training type						
Specific illness	0.51^**^ [0.31–0.84]					
Screening and assessment		0.63^*^ [0.41–0.96]				
Counseling			0.83 [0.56–1.25]			
Psychosocial support				0.67 [0.45–1.01]		
Collaborative work					0.94 [0.64–1.38]	
Law and regulation						0.63^*^ [0.42–0.93]
Model 3, adjusted or age, sex, years of experience, full-time worker status, and community population						
Training type						
Specific illness	0.54^*^ [0.32–0.93]					
Screening and assessment		0.63^*^ [0.39–0.99]				
Counseling			0.76 [0.49–1.19]			
Psychosocial support				0.73 [0.47–1.13]		
Collaborative work					0.98 [0.65–1.48]	
Law and regulation						0.73 [0.47–1.13]

^⁎^ *p* < 0.05

^⁎⁎^ *p* < 0.01

## Discussion

To our knowledge, this is the first study evaluating the efficacy of specific types of mental health training in public health workers in Japan. Among various types of mental health training, training in specific illness and screening and assessment was significantly associated with the sense of knowledge and experience after the adjustment of various potential confounders. These findings are in line with past reports showing significant effects of mental health training on non-specialist workers’ capacities, e.g., knowledge and recognition of mental illnesses [15–20]. The present study may provide information improving the management of mental illness, which is specifically crucial for social recovery in the post-COVID-19 era.

Although our finding is based on the data from a Japanese sample and may not be readily generalizable to other countries, the present study implies that the concept−/assessment-oriented training may enhance public health workers’ capacities. Indeed, the mhGAP intervention guide for non-specialist workers recommends conducting an assessment as an essential clinical practice in mental health [23]. Our finding supports this recommendation from the viewpoint of training efficacy. To note, training in specific illness and screening and assessment may improve knowledge directly related to each mental illness, which may lead to the confidence of public health workers.

Japan has the highest number of psychiatric beds per capita in the world [29], which may co-occur with polypharmacy and long-term hospitalization [30, 31]. Hospital discharge and transition to the communities have been warranted to achieve patient-centered care [32]. To achieve this, the “Reform Vision of Mental Health and Welfare” was released in 2004 [33]. However, Japan still has long-term psychiatric hospitalization (mean, 265.8 days) as of 2019 [34], partly resulting from insufficient community support [22, 35]. The potential approach to improve public health workers’ capacities shown in the present study may have clinical implications in enhancing the quality of community mental health in Japan.

### Limitations

Several limitations should be acknowledged here. First, a lack of knowledge and experience was measured in a subjective manner, which may not reflect the actual capacity of public health workers. It may have been vulnerable to recall and social desirability biases. Second, both a lack of knowledge and experience and mental health training were self-reported via a single-item questionnaire. The cut-off for having a lack of knowledge and experience was arbitrary. Future studies should employ validated measurements to address these issues. Third, this study is based on cross-sectional web-based survey data that does not ascertain the temporal order of events or make causal inferences. Studies with longitudinal data are warranted to understand the causal relationship. Fourth, it is unclear if public health workers’ subjective knowledge and experience result in an improved outcome for patients with mental illness. Finally, our models aimed to adjust for confounding between training types and knowledge and experience rather than to maximize the overall goodness-of-fit, and thus should not be used for prediction.

## Conclusions

Despite these limitations, we believe that the present study provides meaningful information that training in specific illness and screening and assessment may lead to knowledge and experience of public health workers. Further studies should employ a longitudinal design and validated measurements.

## Supplementary Information

**Additional file 1.**

## Data Availability

Not applicable.

## Abbreviations

COVID-19: Coronavirus disease 2019
WHO: World Health Organization
mhGAP: Mental Health Gap Action Programme
