# Supplementary material for: Training types associated with knowledge and experience in public health workers
Source: Arch Public Health. 2022 Jan 27;80:44. doi: 10.1186/s13690-022-00788-4 (PMC8792519; doi:10.1186/s13690-022-00788-4)
Supplement: Supplementary file 1 — Additional file 1. [file 13690_2022_788_MOESM1_ESM.docx]

**Supplementary Table S1 Details of participants’ professional backgrounds. Public health nurses were the largest part of the sample.**

| Professional background, No.^a^ | Overall | Lack of knowledge and experience | |
| --- | --- | --- | --- |
|  |  | Yes | No |
| Public health nurse | 398 | 256 | 142 |
| Psychiatric social worker | 48 | 17 | 31 |
| Social worker (non-psychiatric) | 11 | 7 | 4 |
| Psychologist | 8 | 5 | 3 |
| Physician | 3 | 1 | 2 |
| Nurse | 2 | 2 | 0 |
| Other | 18 | 14 | 4 |

^a^Missing data for seven participants

**Supplementary Table S2 Details of training in specific illness. Schizophrenia was reported most frequently. Twenty-one participants reported Hikikomori, Japanese individuals who intentionally withdraw form society and may have phenomenological characteristics of early psychosis or negative symptoms.**

| Illness, No. | Overall | Lack of knowledge and experience | |
| --- | --- | --- | --- |
|  |  | Yes | No |
| Schizophrenia | 84 | 47 | 37 |
| Autism spectrum disorder | 81 | 41 | 40 |
| Major depressive disorder | 66 | 40 | 26 |
| Alcohol use disorder | 62 | 35 | 27 |
| Personality disorder | 34 | 16 | 18 |
| Hikikomori | 21 | 12 | 9 |
| Bipolar disorder | 10 | 4 | 6 |
